# METAB‐HTX: prospective, longitudinal cohort study evaluating cardiac and systemic metabolism after heart transplantation

**DOI:** 10.1002/ehf2.15330

**Published:** 2025-05-15

**Authors:** Amin Polzin, Daniel Scheiber, Fabian Voss, Jean Haurand, Elric Zweck, Daniel Oehler, Oliver Maier, Mareike Cramer, Maximilian Spieker, Constanze Moos, Ursala Tokhi, David Naguib, Philipp Mourikis, Marcel Benkhoff, Robert Wagner, Michael Roden, Heinz‐Peter Schultheiss, Sascha Dietrich, Hug Aubin, Udo Boeken, Artur Lichtenberg, Malte Kelm

**Affiliations:** ^1^ Department of Cardiology, Pulmonology, and Vascular Medicine, Medical, Faculty of Heinrich Heine University University Hospital Düsseldorf Düsseldorf Germany; ^2^ Cardiovascular Research Institute Düsseldorf (CARID) Heinrich Heine University Düsseldorf Germany; ^3^ Department of Endocrinology and Diabetology Medical Faculty and University Hospital Düsseldorf, Heinrich‐Heine‐University Düsseldorf Düsseldorf Germany; ^4^ Institute for Clinical Diabetology, German Diabetes Center, Leibniz Center for Diabetes Research at Heinrich‐Heine‐University Düsseldorf Düsseldorf Germany; ^5^ German Center for Diabetes Research, Partner Düsseldorf München‐Neuherberg Germany; ^6^ Institute for Cardiac Diagnostics and Therapy (IKDT) Berlin Germany; ^7^ Department of Hematology Heinrich‐Heine University Düsseldorf Germany; ^8^ Department of Cardiac Surgery Heinrich‐Heine University Düsseldorf Germany

**Keywords:** Heart transplantation, Metabolism, Long‐term survival, Diabetes

## Abstract

**Aims:**

Heart transplantation (HTX) is the treatment of choice for advanced heart failure. Still, long‐term survival needs to be improved. Recent studies showed that obesity and type 2 diabetes (T2D) as well as impaired renal and liver function are associated with mortality post‐HTX. There are many open questions including (i) optimal metabolic surveillance post‐transplant, (ii) association of metabolic deterioration and cardiac function, (iii) association with hepatic and renal deterioration, and (iv) optimal timing and choice of treatment. The METAB‐HTX trial will address these open questions, hypothesizing that metabolic deterioration post‐HTX is associated with impaired cardiac function and survival.

**Methods and results:**

METAB‐HTX is a prospective, longitudinal cohort study, enrolling 400 patients post‐HTX in a period of 5 years. Time‐series, deep cardiac, and metabolic phenotyping will be conducted. Cardiac function will be analysed by echocardiography as well as serial cardiac magnetic resonance imaging and spectroscopy (cMRI/MRS). Coronary angiography will be conducted to assess both macrovascular and microvascular coronary allograft vasculopathy (CAV). To evaluate allograft rejection, endomyocardial biopsies will be taken. Metabolic alterations will be investigated by (i) glucometabolic phenotyping including serial oral glucose tolerance tests, homeostasis model assessment, T2D endotyping, and muscle biopsies in selected cases; (ii) lipid disorders will be evaluated by classical lipid measurements in combination with evaluation of HDL function, plasma membrane lipid composition, fluidity analyses of circulating cells and MRI/MRS for adipose tissue distribution, and ectopic fat analysis. Kidney and liver function and structural alterations will be evaluated. Complex analyses will be conducted to evaluate (i) myocardial substrate utilization and energy metabolism by cardiac and circulating cell respirometry, (ii) impact of genetic (including immunogenetic) and transcriptomic factors by third‐ and fourth generation sequencing (short‐ and long‐read sequencing), (iii) circulating signatures of future neoplasia by single‐cell sequencing of circulating leucocytes, and (iv) evaluation of thromboinflammation in association with heart transplant events. The primary endpoint will be the incidence of heart transplant events, defined as worsening of systolic or diastolic left ventricular function, CAV, allograft rejection, worsening of kidney function, metabolic liver disease, infections, neoplasia, deterioration of glucose and lipid metabolism. Secondary outcomes include hospitalizations related to primary endpoints, re‐HTX or ventricular assist device, cardiovascular mortality, and all‐cause mortality.

**Conclusions:**

The METAB‐HTX trial will identify early metabolic alterations potentially impairing cardiac function and outcome of HTX patients. This will identify patients at risk and allow precise planning of interventional trials to treat metabolic alterations post‐HTX and improve outcome.

## Graphical abstract

The METAB‐HTX trial is a prospective cohort study, evaluating cardiac and systemic metabolism among heart transplant recipients. Myocardial function, energetic and metabolism, allograft vasculopathy, allograft rejection, glucometabolism, lipometabolism, liver‐ and kidney function, leucocyte signatures and thromboinflammation will be evaluated longitudinally. The aim is to achieve a better understanding of metabolic changes and identify promising therapeutic targets to improve survival and reduce morbidity in heart transplant recipients.

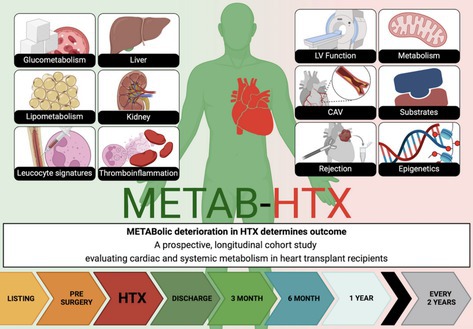

## Introduction

Advanced heart failure occurs in up to 10% of heart failure patients with increasing prevalence.^1^ In these patients, heart transplantation (HTX) improves life expectancy from a median of 12.2 months to 12.2 years.^2^, ^3^ However, there is still a substantial need to improve long‐term survival post‐HTX. To address this need, an in‐depth understanding of factors that lead to worsening of cardiac function and mortality is needed. A recent analysis, investigating 30 606 post‐HTX patients, revealed an association of body mass index, type 2 diabetes (T2D), glomerular filtration rate, and bilirubin concentrations with mortality.^4^ Furthermore, it is known that T2D is frequent after transplantation with an incidence >20% within the first 2 years after transplantation.^5^ This can be partially explained by immunosuppressive therapy post‐HTX that is associated with incidence of T2D.^6^ Hence, it seems likely that metabolic alterations might be a crucial factor after transplantation. However, there are still many open questions. These include (i) an in‐depth understanding of the glucometabolic and lipid alterations over time post‐HTX, (ii) its association with cardiac metabolism and function as well as coronary allograft vasculopathy (CAV), (iii) its association with hepatic disease and renal function, and (iv) optimal timing and choice of treatment. To address these open questions, we conduct the METAB‐HTX trial. We hypothesize that metabolic deterioration post‐HTX is associated with impaired cardiac function and survival. The specific aims of the trial are (i) an in‐depth, time‐series analyses of glucometabolism and lipometabolism post‐HTX; (ii) association of changes in glucose and lipid metabolism with cardiac metabolism, function, CAV, and infections; (iii) association of glucometabolic and lipometabolic changes with hepatic and renal function; (iv) implementation of novel cMRI/MRS techniques to improve post‐transplant surveillance; and (v) evaluate the role of novel leucocyte signatures to predict malignant tumours and CAV as well as thromboinflammation patterns post‐HTX.

This trial will enable an in‐depth understanding of the association of metabolic deterioration, inter‐organ communication, cardiac function, and outcomes post‐HTX. It will pave the road for identification of specific novel therapeutic targets and precise planning and conduction of interventional trials to improve outcome post‐HTX.

## Methods

### Study design

METAB‐HTX is a prospective, longitudinal cohort study. The study will be initiated in the University Hospital Düsseldorf with the option to include further centres (for sub‐studies) to gain extern validation in the future. The study is approved by the Institutional Ethics Committee (Medical Faculty, Heinrich Heine University, Düsseldorf) in accordance with the World Medical Association Declaration of Helsinki. Written consent prior inclusion is required (reference numbers 5263R, 2021‐1635, and 2022‐1962).

### Study population

Patients on the heart transplant waiting list as well as post‐HTX patients will be included. Inclusion criteria will be age >18 years and planned or already conducted HTX. In cases of temporal inability to decide for or against a participation, the patient's legal guardian will be asked for consent provisionally. Exclusion criterion will be the absence of informed consent.

### Study endpoints

**Figure 1:**
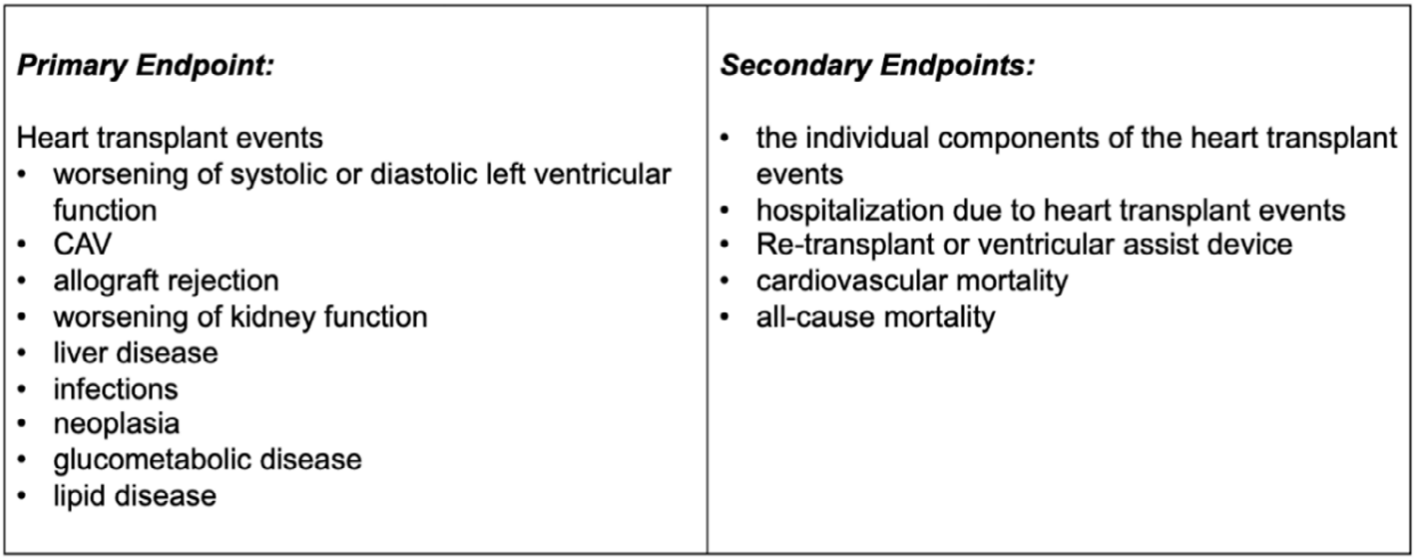
Study endpoints.

The primary and secondary endpoints are summarized in *Figure* *1*. Definitions of individual heart transplant event components are:

Worsening of systolic or diastolic left ventricular function is defined by occurrence or progression of diastolic or systolic dysfunction measured by cMRI or echocardiography. Worsening of systolic function is defined as reduction of LVEF of at least 10% or a relative reduction in GLS of at least 15%. Worsening of diastolic function is defined as new onset of increased grading of diastolic dysfunction according to the guidelines for diastolic function in HTX patients.^7^ Furthermore, any adverse remodelling in myocardial texture and myocardial T1/T2 relaxation times will be documented as heart failure event.

CAV grading will be performed according to the current ISHLT guidelines as previously described, with ISHLT CAV 0 indicating no significant CAV, ISHLT CAV 1 defined as mild CAV, ISHLT CAV 2 as moderate CAV, and ISHLT CAV 3 as severe CAV.^8^, ^9^, ^10^ Every diagnosis of ISHLT CAV > 0 and every progression in CAV grading constitutes a heart transplant event.

Cellular rejection will be defined using the Revised ISHLT Grading Scale, which categorizes rejection from grade 0R (no rejection) to grade 3R (severe rejection) based on the degree of mononuclear cell infiltration and associated myocyte damage in endomyocardial biopsies. Humoral rejection, or antibody‐mediated rejection (AMR), is evaluated based on histopathologic and immunopathologic criteria, including evidence of microvascular injury, complement deposition (C4d staining), and circulating donor‐specific antibodies (DSA). AMR is classified as pAMR 0 (no AMR), pAMR 1 (suspected AMR), or pAMR 2 (definite AMR) according to biopsy findings and serologic markers. Any rejection diagnosis of >ISHLT G 0R or >pAMR 0 constitutes a heart transplant event.

Worsening of kidney function will be determined by eGFR mean slope change [mL/min/1.73 m^2^] as well as classification of acute kidney injury according to KDIGO criteria (creatinine increase 0.3 within 48 h/1.5‐fold change within 7 days).

Liver disease will be defined by occurrence of structural liver disease according to current guidelines^11^ or increase of liver plasma enzyme concentrations.

Infections requiring healthcare professional interventions will be defined as heart transplant event.

Neoplasia is defined as first diagnosed and histopathological confirmed solid or non‐solid malignant tumours.

Glucometabolic disease will be defined as (i) impaired glucose tolerance (2‐h glucose levels of 140–199 mg per dL on the 75‐g oral glucose tolerance test), (ii) impaired fasting glucose (fasting plasma glucose values of 100–125 mg per dL), or (iii) diagnosis of T2D according to current criteria of the American Diabetes Association.^12^

Lipid disease is defined as (i) elevating low‐density lipoprotein concentrations, apolipoprotein B concentrations, and fasting triglyceride levels; (ii) impaired HDL function; (iii) enhanced membrane fluidity; or (iv) membrane cholesterol enrichment in time‐series analyses.

### Measurements

**Figure 2:**
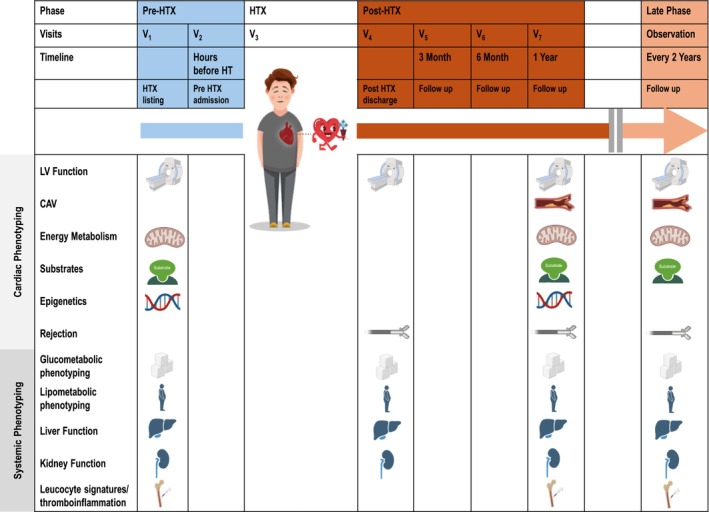
Study visits and procedures.

Study visits and procedures per visit are summarized in *Figure* *2*. A detailed overview of measurements is attached in *Table* *1*.

**Table 1:** Study protocol

			V_1_	V_2_	V_3_	V_4_	V_5_	V_6_	V_7_	V_8_	V_x_
			Listing	Pre‐HT	Post‐HT	3M	6M	1Y	3Y	5Y	1/Y
General information	Inclusion/exclusion criteria		x								
	Informed consent form		x								
	Demographics		x								
	Medical history		x	x	x	x	x	x	x	x	x
	Medication		x	x	x	x	x	x	x	x	x
	KCCQ		x					x	x	x	
	Vital signs		x			x	x	x	x	x	
	ECG		x			x	x	x	x	x	
Cardiac metabolism	Diastolic and systolic LV—dysfunction	TTE	x					x	x	x	x
		RHC	x					x	x	x	
		MRI	x					x	x	x	x
		Hs troponin T	x			x	x	x	x	x	x
		Nt‐proBNP	x			x	x	x	x	x	x
	Macrovascular and microvascular complications of CAV	Angiography + IVUS/OCT	x					x	x	x	x
		FFR/CFR/MRR						x	x	x	
	Cardiac energy formation and consumption	CPX	x					x	x	x	
	Substrate metabolism and mitochondrial function	Respirometry						x	x	x	
	(Epi)genetics Transcriptomics	Leucocytes	x					x	x	x	
		Myocardium						x		x	
	Inflammation and allograft rejection	EMB						x	x	x	
		CRP	x		x			x	x	x	
Systemic metabolism	2‐h OGTT		x		x			x	x	x	
	Hyperglycaemia and T2D	Insulin	x		x			x	x	x	
		C‐peptide	x		x			x	x	x	
		FFA	x		x			x	x	x	
		FPG	x		x			x	x	x	
		RRPG	x		x			x	x	x	
		HbA1c	x		x			x	x	x	
		HOMA‐IR/beta	x		x			x	x	x	
		OGIS	x		x			x	x	x	
		PREDI‐M	x		x			x	x	x	
		T1D‐specific antibodies	x								
	Insulin resistance and metabolic syndrome	BMI	x		x			x	x	x	
		Waist–hip–ratio	x		x			x	x	x	
		Total Cholesterol	x		x			x	x	x	
		TG	x		x			x	x	x	
		HDL	x		x			x	x	x	
		LDL	x		x			x	x	x	
	AST/ALT		x		x			x	x	x	
	Liver function	GGT	x		x			x	x	x	
		AP	x		x			x	x	x	
		Bilirubin (direct/indirect)	x		x			x	x	x	
		INR	x		x			x	x	x	
		Antithrombin	x		x			x	x	x	
		Ammonia	x		x			x	x	x	
		Cholinesterase	x		x			x	x	x	
		Serum ferritin	x		x			x	x	x	
		Fib‐4 score	x		x			x	x	x	
	Kidney function	Urinalysis	x		x			x	x	x	
		eGFR	x		x			x	x	x	
		Albuminuria	x		x			x	x	x	
	Bone marrow	BM aspiration	x					x			
		Complete blood count	x		x			x	x	x	
	Intestine and microbiome	Faecal sample	x					x	x	x	

### Cardiac phenotyping

Left ventricular function will be measured by cMRI (3.0 T Ingenia Elition, PHILIPS Healthcare, Best, Netherlands), including measurements of volume, mass, function (systolic and diastolic), perfusion, and myocardial relaxometry. Feature Tracking analysis and 4D‐Flow measurements will be performed.^13^ Left‐ and right ventricular end‐diastolic and end‐systolic frames will be identified from short‐axis steady‐state free‐precession (SSFP) cine images with whole ventricle coverage from base to apex. LV mass, LV and right ventricular end‐diastolic and end‐systolic volumes, with the corresponding ejection fractions will be calculated using the Simpson disc summation method. Four‐, three‐, and two‐chamber cine views will be used to measure left and right atrial volume and function. Additionally, native relaxometric mapping of the myocardium (T1, T2, T2*), gadolinium contrast‐enhanced imaging (LGE), and post‐contrast relaxometric mapping (T1, extracellular volume) will be performed to assess myocardial inflammation, extracellular volume, and fibrosis. Dual‐sequence perfusion will be acquired following either vasodilator administration (regadenoson) or ergometer physical exercise. Myocardial perfusion images will be acquired at basal, mid‐ventricular, and apical levels in short‐axis slices, allowing automated pixel‐wise myocardial blood flow mapping and assessment of myocardial perfusion reserve for each of the 16 American Heart Association segments shown as a bull's‐eye plot. Quantitative perfusion will be performed as well. Certified CMR evaluation software will be used for all analyses (CVI42, Circle Cardiovascular Imaging Inc., Calgary, Alberta, Canada, or Sectra Workstation IDS7, Version 19.3.6.3510). For ^1^H MRS measurements, spectra will be acquired in the interventricular septum using a point‐resolved spectroscopy sequence (PRESS) with reduced spoiler areas and a spectral BW of 2000 Hz (1024 samples).^14^, ^15^ Metabolite cycling is implemented by adding the optimized Hwang pulse in front of the sequence.^16^ Spectra will be reconstructed in MATLAB using a customized reconstruction pipeline implemented in ReconFrame (GyroTools LLC, Zurich, Switzerland). Spectra will be fitted in AMARES to obtain CR/W and TG/W ratios.^17^, ^18^ If cMRI is not possible due to patient‐related factors, echocardiography will be applied. We have already tested comparability of different methods in the past.^19^

Coronary angiography together with intravascular imaging and intravascular physiology measurements will be conducted to diagnose CAV. Intracoronary imaging will be applied to identify intimal thickening of the coronary arteries.^20^ Optical coherence tomography (OCT) offers the highest imaging quality^21^ but requires contrast agent application and will thus be applied in patients with an estimated glomerular filtration rate (eGFR) > 30 mL/min using a Dragonfly™ OPTIS™ catheter (Abbott Laboratories, Illinois; USA). Patients with an eGFR <30 mL/min will be evaluated by intravascular ultrasound (IVUS) using an Eagle Eye Platinum catheter (Koninklijke Philips N.V., Amsterdam, The Netherlands). Diagnosis of coexisting or isolated microvascular disease will be achieved through intravascular physiologic assessment. Fractional flow reserve (FFR) analysis will be performed using a pressure‐sensitive guidewire. FFR is an index of the functional relevance of a coronary stenosis and is defined as the ratio of mean distal coronary pressure to mean aortic pressure at maximal hyperaemia. Following FFR, we will use a thermodilution‐based approach to determine coronary flow reserve (CFR), which is an index of coronary blood flow at rest and hyperaemic conditions and is considered a marker of microvascular disease.^22^ FFR and CFR measurements will be performed using a PressureWire™ X Guidewire (Abbott Laboratories, Illinois, USA). By dividing CFR by FFR while adjusting for aortic pressure, we will be able to calculate the microvascular resistance reserve (MRR), which is a recently developed tool for assessing the coronary vasodilatory capacity while correcting for both macrovascular coronary artery disease and vasodilator use.^23^

Allograft rejection will be evaluated by endomyocardial biopsy. Tissue specimens will be collected from the interventricular septum under standard fluoroscopic guidance for histochemical analysis at different time points post‐HTX. Endomyocardial tissue specimens will be analysed by cardiac transplant pathologists in accordance with the International Society for Heart and Lung Transplantation (ISHLT) criteria.^20^ Immunohistochemistry will be conducted to characterize inflammatory infiltrates, followed by quantitative digital image analysis. CD3+ lymphocytes will be identified using a CD3 antibody (Dako; dilution 1:25). Myocardial inflammation will be quantified by assessing CD11a+/lymphocyte function‐associated antigen (LFA)‐1+ lymphocytes/mm^2^, CD11b+/Mac‐1+ macrophages/mm^2^, CD45R0+ T memory cells (Dako, Glostrup, Denmark), and perforin+ cytotoxic cells/mm^2^. All antibodies will be procured from ImmunoTools (Friesoythe, Germany) and BD Biosciences (San Jose, California). An EnVision peroxidase‐conjugated anti‐mouse antibody (DakoCytomation) will serve as the secondary antibody, and immunohistochemical staining will be visualized using 3‐amino‐9‐ethylcarbazole (Merck, Darmstadt, Germany) as the chromogenic substrate. Slides will be counterstained with haematoxylin and mounted using Kaiser's gelatine R (Merck). Quantification of immunoreactivity will be performed through digital image analysis at 200× magnification, with results reported as the number of positive cells per square millimetre (mm^2^).

Furthermore, the Kansas City Cardiomyopathy Questionnaire (KCCQ) will be conducted to assess health status over time. KCCQ is a self‐administered, 23‐item instrument evaluating symptoms, physical function, social limitations, and quality of life, providing a comprehensive view of the patient's condition as described before.^24^ Participants will complete the questionnaire at multiple time points to track changes in their health status throughout the study. Cardiopulmonary exercise testing (CPET) will be employed to evaluate the cardiorespiratory function and exercise capacity of HTX patients. Participants will undergo a graded exercise test on a cycle ergometer with continuous measurement of respiratory parameters, including oxygen consumption (VO_2_), carbon dioxide production (VCO_2_), and ventilation (VE), utilizing a metabolic cart as described before using a Metalyzer® 3B CORTEX Biophysik GmbH (Leipzig, Germany).^25^ Additionally, myocardial mitochondrial metabolism will be assessed using high‐resolution respirometry (HRR) in permeabilized myocardial fibres as described previously.^26^, ^27^ In short, scar‐free myocardial tissue will be isolated, permeabilized with saponin, and prepared in a respiration medium. Single fibres will be weighed and placed in Oxygraph chambers for oxygen flux measurements at 37°C. Using predesigned protocols, various substrates and inhibitors, such as malate, octanoyl‐carnitine, ADP, glutamate, succinate, cytochrome C, oligomycin, FCCP, and antimycin A will be sequentially applied to assess different states of respiration, including coupled (P), uncoupled (E), and leak respiration (L).^28^

Total RNA from human heart biopsies will be isolated and used for library preparation following manufacturer protocols. HiFi/CCS sequencing will be performed using SMRTbell library preparation (PacBio) and analysed with SMRT Link v9.0. Long‐read sequencing will be conducted at the BMFZ, Heinrich Heine University Düsseldorf, using the Revio System. Short‐read RNA sequencing will use DNase‐digested RNA, quantified via Qubit and Fragment Analyser (Agilent). Libraries will be prepared using the VAHTS™ RNA‐Seq Kit for Illumina® and sequenced on HiSeq 3000/4000 (Illumina). Fastq files will be processed with bcl2fastq for trimming and demultiplexing. Long‐read data will construct a disease‐specific transcriptome, against which short‐read data will be mapped. Comparisons will be made to the GRCh38 reference transcriptome. Principal component analysis will assess mapping effects, incorporating clinical variables. Fastq analyses will be performed using CLC Genomics Workbench, with reads trimmed for quality and adapters.

### Systemic phenotyping

Kidney function will be assessed through periodic blood and urine samples, which will be analysed for renal function parameters. These include serum creatinine and cystatin c for assessment of eGFR, blood urea nitrogen (BUN), and glutamic acid decarboxylase antibodies (GADA, IA2A, ZnT8, mIAA). Additionally, proteinuria (urinary albumin and creatinine) will be measured as a key indicator of kidney injury. This allows a precise staging of chronic kidney disease according to KDIGO criteria.^29^ As part of the extensive pre‐transplant screening, as well as in the case of adverse renal‐events, like AKI, duplex sonography of the kidney provides information on morphological and functional characteristics. The renal resistive index is measured as a direct marker of renal blood flow, which can also depict the complex interplay between systemic circulation and renal microcirculation. It also correlates with renal arteriosclerosis and CKD.^30^ As a prognostic factor in cardiovascular disease and after HTX, renal outcome will be additionally observed in the context of unavoidable HTX‐specific renal impacts, including immunosuppressive regimens with calcineurin inhibitors.^31^

Liver function in HTX patients will be evaluated through periodic blood samples, including alanine aminotransferase, aspartate aminotransferase, alkaline phosphatase, total bilirubin, and albumin levels. Additionally, the international normalized ratio will be measured to assess coagulation function. Present or history of alcohol consumption (>30 g/day in men or >20 g/day in women) is rigorously screened for in HTX screening and renders individuals ineligible for HTX. All patients undergoing transplantation screening undergo abdominal computer tomography and abdominal sonography including transient elastography to rule out advanced fibrosis or cirrhosis. As part of the present study protocol, after transplantation, HTX recipients will repeatedly undergo repeated screening for hepatic fibrosis using a stepwise approach in accordance with current guidelines^11^: An initial screening will be performed using the FIB‐4 score at every visit. Participants with a FIB‐4 score above 2.67 (above 2.0 in patients older than 65 years) will be referred to hepatology for transient elastography to screen for metabolic dysfunction‐associated steatotic liver disease (MASLD).

Neoplasia will be screened as proposed by the ISHLT guidelines for post‐HTX care. Before HTX all patients undergo in‐depth screening for occult malignancies by whole‐body CT scans, screening for occult blood within the stool, and if indicated gastroscopy and coloscopy. Additionally, screening for dermal malignancies is performed by board‐certified dermatologists and urologists, and in female patients, gynaecological malignancies are excluded by urologists and gynaecologists. After HTX patients are at least annually screened for dermal malignancies by a dermatologist. Malignoma screening by urologists and gynaecologists is performed annually. Additionally, abdomen sonography and chest x‐ray are performed annually to screen for solid malignomas and lymph node enlargements. Gastroscopy and coloscopy are performed as indicated by detection of occult blood in stool or every 5 years after the age of 50.

Glucometabolic phenotyping will be conducted by measuring haemoglobin A_1c_ levels, fasting blood glucose, and 2‐h glucose above 200 mg/dL during an oral glucose tolerance test. Patients will be screened for diabetes‐associated antibodies to rule out type 1 diabetes (GAD65 [GAD] autoantibodies, IA‐2 [IA‐2] autoantibodies, zinc transporter 8 [ZnT8] autoantibodies, islet cell autoantibodies, insulin autoantibodies). In participants without insulin or sulfonylurea therapy with fasting blood glucose below 140 mg/dL, a 3‐h OGTT will be performed with blood sampling every 30 min after 75 g glucose ingestion. In addition, circulating insulin, c peptide, and free fatty acid levels will be measured in all patients. Insulin resistance will be assessed from the oral glucose insulin sensitivity index (3‐h OGIS) and the predicted M‐value (PREDIM).^32^ Additionally, insulin resistance will be estimated based on the homeostasis model assessment (HOMA‐2‐IR).^33^ In participants with confirmed diabetes mellitus, endotyping will be conducted in accordance with previous studies based on the clusters identified by Ahlqvist *et al*.^34^ and externally validated by other groups.^35^ Additionally muscle biopsies will be performed in selected cases to determine energy metabolism and inflammation within the skeleton muscle in diabetic and non‐diabetic transplant recipients.

Lipometabolic phenotyping will be conducted by time‐series measurement of circulating lipid levels (HDL, LDL, apolipoprotein B, triglycerides). In selected cases, MRI/MRS will be performed to analyse fat tissue distribution and detect ectopic fat deposits. Additionally, HDL function and quality will be determined via cholesterol efflux capacity assay (Abcam, USA). The influence of circulating lipids on the blood cells is also determined. The composition of the membrane of leucocytes and platelets, including the cholesterol content, is determined using liquid chromatography–tandem mass spectrometry (LC‐MS/MS) measurements. The resulting changes in membrane fluidity are analysed after incubation with fluorescent probes TMA‐DPH and DPH via measurement of anisotropy using a spectrophotometer (PerkinElmer, USA).

Additionally, leucocyte signatures will be determined. We will use flow cytometry for an initial assessment of leucocyte activation. Next, we will perform single‐cell sequencing to get deeper insights into leucocyte status over the time course. In addition, we will analyse epigenetic alterations in leucocytes. This will allow identification of certain leucocyte subsets which are associated with post‐HTX events like neoplasia and build the base for following mechanistic trials.

Platelet reactivity and thromboinflammation will be measured by different assays. First, circulating citrullinated histone H3 (H3cit)‐DNA complexes will be measured as previously described.^36^ This is a validated marker for circulating neutrophil extracellular trap (NET) formation. Second, we will investigate the interaction between platelets and leucocytes (especially neutrophils) via flow cytometry. Third, platelet reactivity in general will be analysed with flow chamber assays (BioFlux 200, I&L Biosystems GmbH, Germany), degranulation assays (Merck, Germany), and light transmission aggregometry (APACT 4004, LABiTec, Germany).

Medications, especially immunosuppressant agents, and their serum levels will be analysed during every visit (*Table* *1*). A standardized treatment protocol for immunosuppression post‐HTX minimizes the chance of bias by different immunosuppression regimes. Nevertheless, future multicenter sub‐studies may help to compare different immunosuppressant agents and their metabolic consequences. Dependency of glucometabolic and lipometabolic changes on medications will be analysed longitudinally.

### Sample size calculation

This study will prospectively include all new transplanted HTX recipients at our centre. We estimate 50 new transplantations per year. Additionally, we aim to recruit previously transplanted patients currently receiving follow‐up care at our centre. This cohort consists of 250 living HTX recipients. Based on an expected participation rate of 90%, we anticipate enrolling approximately 270 patients in the first year of the study. To address the initial hypothesis of the trial, metabolic deterioration post‐HTX is associated with impaired cardiac function and survival.

Based on previous studies, this sample size will yield a sufficient number of outcome events during the follow‐up of at least 100. Exact events rates based on glucometabolic disease are difficult to estimate from previous studies in this highly selected population, as neither glucometabolic disease nor respective outcomes have been as systematically or extensively screened for in previous trials. Thus, we estimate that, with a sample size of 400, at an alpha level of 0.05, assuming that 60% of patients develop glucometabolic disease after HTx and further assuming that the hazard for the primary outcome events is 35% higher (hazard ratio 1.35) in patients with glucometabolic disease, the estimated power for this study would be 0.8090 which is above the target of 0.8.

Beyond that, the trial is designed to allow further sub‐studies and even nested RCTs to address the aim of the study to achieve an in‐depth analyses of the interaction between metabolism, cardiac function, and inter‐organ communication and to reveal and test novel therapeutic regimens.

## Discussion

Long‐term survival post‐HTX needs to be improved. In‐depth analyses of underlying causes to develop specific diagnostics and therapy are urgently needed. T2D is frequent in post‐HTX^37^ patients. This is partially explained by immunosuppression.^6^ Furthermore, T2D has been shown to be a risk factor for mortality in post‐HTX patients.^38^ However, not only T2D but early glucometabolic changes might already impair cardiac function. We have previously shown that insulin resistance is already associated with decreased myocardial mitochondrial efficiency and integrity. This resulted in severe myocardial dysfunction in mice undergoing acute myocardial infarction.^39^ Indeed, we were able to show in human heart biopsies that impaired mitochondrial oxidative capacity was associated with impaired cardiac function.^40^ Furthermore, we found that myocardial mitochondrial capacity was already reduced in mild cellular rejection of the transplanted heart.^41^ In line, mechanical cardiac unloading reduced myocardial mitochondrial reactive oxygen species production.^27^ In patients with T2D and apparently healthy hearts, ventricular mitochondrial function was already impaired. This was associated with myocardial oedema, early signs of impaired myocardial contractility, and diastolic function.^42^ Importantly, we found that in heart samples from apparently healthy human, impaired glucose tolerance was associated with impaired mitochondrial respiration in human ventricular myocardium.^42^ Hence, a detailed understanding of glucometabolic alterations in post‐HTX patients is urgently needed to prevent subsequent impairment of cardiac function. Further, in‐depth analyses of myocardial energy metabolism will identify patients at risk and might be a promising therapeutic target in the future. Besides glucometabolism, this trial will focus on lipometabolic phenotyping of post‐HTX patients. It is well known that dyslipidaemia is associated with mortality. Strict therapeutic goals and regimen are established for the general population and patients with cardiovascular disease.^43^ Post‐HTX, statins have been shown to improve survival and reduce allograft rejection and CAV.^44^ Even first case series of proprotein convertase subtilisin/kexin type‐9 inhibitors show safety and efficacy post‐HTX.^45^ Furthermore, first studies show that addressing cholesterol efflux may reduce heart transplant events.^46^ We have previously shown that not high‐density lipoprotein concentrations but their function is crucial in prevention of adverse events. Furthermore, we found that bioactive lipid content of HDL is associated with its protective functions.^47^ This lipoprotection has important implications in various cardiovascular diseases.^48^, ^49^, ^50^ Still, many open questions remain in post‐HTX patients including the impact of lipometabolic alterations on (i) cardiac energy metabolism, (ii) cardiac function, (iii) microvascular CAV, and (iv) optimal cut‐offs to initiate or intensify treatment. These explorative analyses will build the basis for further mechanistic investigations to clarify causal relations and therefore to distinguish the contributions of different metabolic pathways to adverse outcomes. Furthermore, renal function will be monitored closely in this trial. It has been shown that kidney disease is a risk factor for mortality post‐HTX.^51^ We have previously identified risk factors of kidney injury in HTX patients with previously preserved renal function.^52^ In METAB‐HTX, we will closely monitor kidney function including markers beyond glomerular filtration rate. We will associate kidney function with cardiac function, energy metabolism, and heart transplant events. Furthermore, we previously investigated the impact of kidney disease on platelets.^53^, ^54^ Platelets mediate thromboinflammation. We have recently shown the role of thromboinflammation in acute myocardial infarction and stroke.^55^, ^56^, ^57^ However, the role of thromboinflammation post‐transplant is completely unknown and will be investigated in this trial. Besides platelets, we have profound expertise on leucocyte signatures that might predict disease.^58^ These signatures in prediction of CAV and neoplasia will be investigated post‐HTX. Additionally, we and others could show that in order to understand specific disease signatures, it is necessary to capture the organ‐specific, disease‐state‐specific full‐length transcriptome.^59^, ^60^ This approach enables the identification of a mRNA transcriptome with 99.9% sequencing accuracy, as entire transcripts can be sequenced in full length, rather than being fragmented and reassembled based on a non‐organ‐specific, non‐disease‐specific reference, as in short‐read sequencing. This specific disease signature will be utilized in combination with classical short‐read sequencing to enable a quantitative analysis of both known and potentially newly discovered transcripts. By integrating the qualitative approach—establishing a disease‐state‐specific reference map—with the quantitative power of short‐read sequencing, this study provides an unprecedented opportunity for a comprehensive molecular characterization post‐HTX.

Regarding hepatic disease, we have shown that MASLD is associated with insulin resistance and impaired mitochondrial efficiency and cardiac function after acute myocardial infarction.^39^ This goes in line with clinical data associated hepatic function and mortality post‐HTX.^61^ Hence, this will be closely monitored in METAB‐HTX as well. We have a long‐lasting experience with novel cMRI sequences and methods. Recently, we revealed quantitative assessment of inflammation using fluorine imaging.^62^ cMRI is promising in post‐HTX surveillance.^63^, ^64^ Therefore, serial cMRI/MRS measurements will be performed in the trial, combining classical measurements as well as novel sequences and techniques.

Taken together, METAB‐HTX will allow a comprehensive analysis of the association of (a) glucometabolic and lipometabolic changes with (b) heart transplant events and (c) inter‐organ interaction with liver and renal deterioration. A combination of classical parameters as well as novel concepts including myocardial energy metabolism, novel cMRI/MRS analyses, cholesterol efflux and HDL function, leucocyte signatures, and thromboinflammation will be applied. METAB‐HTX will pave the road for specific novel marker and target identification as well as optimal planning of interventional trials to improve survival post‐HTX. It will allow further sub‐studies as well as nested‐randomized controlled trials.

## Conflict of interest

None declared.

## Funding

This work was supported by the Deutsche Forschungsgemeinschaft (DFG, German Research Foundation) grant no. 530690968 to M.B.; grant no. 236177352‐SFB1116; grant no. TP B11 to A.P.; grant nos. TP B6 and B12 to M.K. and M.R.; and grant nos. 493400536, 413659045, and 510844896 to A.P. This work was also supported by the German Heart Foundation, grant no. F/32/22 to. A. P, and by the Medical Faculty of the Heinrich Heine University (No. 18‐2019 to A.P. and No. 2021‐01 to P.M.).
